# Crystal chemistry and photomechanical behavior of 3,4-dimethoxycinnamic acid: correlation between maximum yield in the solid-state topochemical reaction and cooperative molecular motion

**DOI:** 10.1107/S2052252515017297

**Published:** 2015-10-16

**Authors:** Manish Kumar Mishra, Arijit Mukherjee, Upadrasta Ramamurty, Gautam R. Desiraju

**Affiliations:** a Solid State and Structural Chemistry Unit, Indian Institute of Science, Bangalore 560 012, India; b Division of Molecular Imaging and Photonics, Department of Chemistry, KU Leuven, Celestijnenlaan 200 F, B-3001 Leuven, Belgium; c Department of Materials Engineering, Indian Institute of Science, Bangalore 560 012, India; d Centre for Excellence for Advanced Materials Research, King Abdulaziz University, Jeddah 21589, Saudi Arabia

**Keywords:** crystal engineering, cinnamic acid, photosalient, nanoindentation, polymorphism

## Abstract

A new monoclinic polymorph, form II (*P*2_1_/*c*, *Z* = 4), has been isolated for 3,4-dimethoxycinnamic acid (DMCA). Its solid-state 2 + 2 photoreaction to the corresponding α-truxillic acid is different from that of the first polymorph, the triclinic form I (, *Z* = 4) that was reported in 1984. The crystal structures of the two forms are rather different. The two polymorphs also exhibit different photomechanical properties. Form I exhibits photosalient behavior but this effect is absent in form II. These properties can be explained on the basis of the crystal packing in the two forms. The nanoindentation technique is used to shed further insights into these structure−property relationships. A faster photoreaction in form I and a higher yield in form II are rationalized on the basis of the mechanical properties of the individual crystal forms. It is suggested that both Schmidt-type and Kaupp-type topochemistry are applicable for the solid-state *trans*-cinnamic acid photodimerization reaction. Form I of DMCA is more plastic and seems to react under Kaupp-type conditions with maximum molecular movements. Form II is more brittle, and its interlocked structure seems to favor Schmidt-type topochemistry with minimum molecular movement.

## Abstract 2

A new monoclinic polymorph, form II, of 3,4-dimethoxycinnamic acid has been isolated and shows a different photochemical and photomechanical property from the previously reported triclinic form I. The solid-state 2 + 2 photodimerization of these polymorphs is rationalized on the basis of minimum and maximum molecular movement during the reaction – the so-called Kaupp and Schmidt models for these reactions.

## 1. Introduction   

Mechanical properties of molecular solids have attracted considerable recent attention in crystal engineering (Desiraju, 2013 ▸; Desiraju *et al.*, 2011 ▸; Varughese *et al.*, 2013 ▸) because of their potential in terms of applications such as modification of hardness, *H*, of active pharmaceutical ingredients (Mishra *et al.*, 2014 ▸, 2015*b* ▸; Sanphui *et al.*, 2015 ▸; Karki *et al.*, 2009 ▸), and in the design of actuators, sensors and other memory devices (Terao *et al.*, 2012 ▸; Balzani *et al.*, 2000 ▸; Kobatake *et al.*, 2007 ▸; Fletcher *et al.*, 2005 ▸; Lv *et al.*, 2010 ▸; Morimoto & Irie, 2010 ▸; Fratzl & Barth, 2009 ▸). The mechanical behavior of these organic and metal–organic solids is often related to their crystal packing (Varughese *et al.*, 2013 ▸; Reddy *et al.*, 2010 ▸; Ghosh & Reddy, 2012 ▸; Ghosh, Mishra, Kadambi *et al.*, 2015 ▸; Mukherjee & Desiraju, 2014 ▸; Tan & Cheetham, 2011 ▸). The variegated types of mechanical responses that have been observed so far include bending, shearing, twisting and jumping (Morimoto & Irie, 2010 ▸; Reddy *et al.*, 2010 ▸; Ghosh, Mishra, Ganguly *et al.*, 2015 ▸; Sahoo *et al.*, 2013 ▸; Panda *et al.*, 2014 ▸, 2015 ▸; Zhu *et al.*, 2011 ▸, 2014 ▸; Medishetty *et al.*, 2014 ▸, 2015 ▸; Nath *et al.*, 2014 ▸; Kim *et al.*, 2014 ▸; Shtukenberg *et al.*, 2014 ▸). Although these types of mechanical responses are most often caused by the inherent anisotropy of crystal packing, they can sometimes be triggered by external perturbations such as photoinduced reactivity (Morimoto & Irie, 2010 ▸; Terao *et al.*, 2012 ▸; Medishetty *et al.*, 2014 ▸, 2015 ▸; Nath *et al.*, 2014 ▸; Kim *et al.*, 2014 ▸). Although many types of photoinduced reactions in solids are known, 2 + 2-type cycloaddition reactions in alkenes are the most often studied. The various mechanical effects that result from this type of transformation are usually explained on the basis of strain generation within the crystals (Zhu *et al.*, 2011 ▸, 2014 ▸; Medishetty *et al.*, 2014 ▸, 2015 ▸; Nath *et al.*, 2014 ▸; Kim *et al.*, 2014 ▸).

While considering strain that is built up within a crystal in the course of a 2 + 2 photoreaction, the widely explored *trans*-cinnamic acids pose an interesting problem. It is now more than 50 years since Schmidt and co-workers in the Weizmann Institute published a series of seminal papers on the topochemical 2 + 2 photodimerization reactions of substituted *trans*-cinnamic acids (Cohen & Schmidt, 1964 ▸; Cohen *et al.*, 1964 ▸; Schmidt, 1964 ▸). Over the years, different aspects of this prototype solid-state reaction, which has also been extended to other alkenes, were discussed in several different publications (Georgiev & MacGillivray, 2007 ▸; Nagarathinam *et al.*, 2008 ▸; Fonseca *et al.*, 2008 ▸; Biradha & Santra, 2013 ▸; Khoj *et al.*, 2013 ▸; Telmesani *et al.*, 2015 ▸). Schmidt’s thesis is that for a photodimerization of this type to occur, the mid-points of the ‘potentially reactive’ double bonds must be within a threshold distance of each other, around 4.0 Å (Schmidt, 1971 ▸). Implicit in this straightforward topochemical model is that the chemical reaction occurs with a minimum of molecular movement. Therefore, the topology of molecules in the reactant is fully retained in the stereochemistry of the product. However, several mechanistic issues crop up that include the fact that the experimental yield can be considerably less than the theoretical yield (Cohen *et al.*, 1964 ▸; Desiraju & Kannan, 1986 ▸; Ramamurthy & Venkatesan, 1987 ▸), or that reactions can take place even when the distance between the reactive molecules is more than the permitted limit (Kaupp & Zimmermann, 1981 ▸; Cohen, 1975 ▸; Kearsley & Desiraju, 1985 ▸; Nakanishi *et al.*, 1985 ▸; Kaupp, 1996 ▸). Sometimes, the reaction does not take place even when the relevant distance is below the threshold value (Nakanishi *et al.*, 1985 ▸; Kaupp, 1996 ▸). All these observations indicate that other factors are important and point to the possibility of ‘surface reactions’ that were described in detail by Kaupp who used AFM to show that surface reactions are a reality in the cinnamic acid system and that ‘substantial material transport’ was taking place during the solid-state transformations (Kaupp, 1992*a* ▸,*b* ▸). Kaupp, indeed, has outlined several situations in which exceptions to Schmidt’s topochemical postulate are possible, leading one to the consideration of cases in which gross molecular movement in the solid state is facile. The kernel of Kaupp’s postulate lies in the formation of slip planes (usually determined by the weaker interactions) in the crystal structures that facilitate the molecular movements within a crystal (Kaupp & Naimi-Jamal, 2005 ▸; Kaupp *et al.*, 2002 ▸). In a nutshell, these two differing views lay their bases on ‘minimum’ *versus* ‘maximum’ movements within crystals that are related to the crystal packing. The problem with cinnamic acid 2 + 2 photodimerization is therefore stated simply enough: does the reaction take place with minimum or maximum molecular movement? Accordingly, any quantitative way to detect changes in crystal packing would be helpful to study such systems.

It is only recently that nanoindentation has emerged as a reliable technique to study the mechanical properties of molecular crystals (Varughese *et al.*, 2013 ▸; Mishra *et al.*, 2013 ▸, 2015*a* ▸; Mishra, Desiraju *et al.*, 2014 ▸; Ramamurty & Jang, 2014 ▸; Chattoraj *et al.*, 2014 ▸; Mishra *et al.*, 2015*c* ▸). Particularly, it has been shown to be useful in the investigation of structure–property relationships in organic crystals, for example the relationships between elastic modulus, *E*, and melting points in α,ω-alkanedicarboxylic acids (Mishra *et al.*, 2013 ▸) and between hardness, *H*, and solubility in curcumin and sulfathiazole polymorphs (Mishra *et al.*, 2014 ▸). To the best of our knowledge, the utility of this technique has not yet been explored to rationalize photomechanical properties that are consequent upon photoirradiation, such as photosalience (Zhu *et al.*, 2014 ▸). With this background, we attempted to study the crystal chemistry of 3,4-dimethoxycinnamic acid (DMCA) with the aid of the nanoindentation technique.

**Figure 1:**
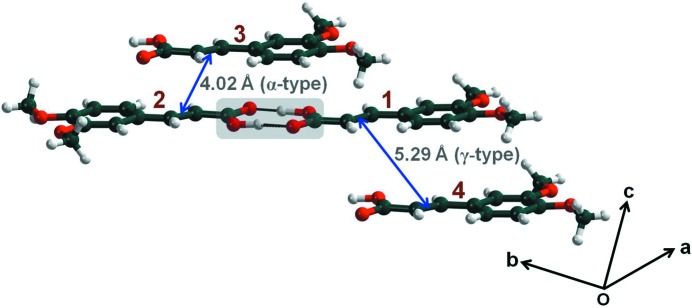
Crystal packing of form I of DMCA. Note that molecules 1 and 4 are in a γ-type environment, while molecules 2 and 3 (translationally related to 4) are in the α-type environment and potentially reactive upon irradiation.

In 1984, Desiraju and coworkers reported a triclinic modification (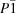, *Z* = 4), of DMCA that was obtained by crystallization from 1:1 MeOH–acetone (Desiraju, Kamala *et al.*, 1984 ▸). The crystal structure is unusual for a cinnamic acid in that while one of the two symmetry independent molecules is situated in the α-type environment (the nearest neighbor is inversion related and within photoreactive distance), the other is in a γ-type environment (the nearest neighbor is pseudo-translated and beyond the 4 Å reacting threshold distance; Fig. 1 ▸). A better resolved crystal structure of this form was published in 1989 but the packing features are essentially the same as that reported previously (Desiraju *et al.*, 1991 ▸). In effect, only half of the molecules in the crystal are potentially reactive in the solid state according to Schmidt’s criterion, and these are the ones that are related by the inversion center. The maximum yield in the photodimerization reaction of this triclinic form was observed to be around 40%, and this was taken to be a confirmation of the topochemical argument by Desiraju, Kamala *et al.* (1984 ▸). However, and as noted for the α-acids studied by Schmidt, the maximum yield is not 50%, which is what might have been expected under strictly topochemical conditions, but lower (Cohen *et al.*, 1964 ▸). This original form is hereafter referred to as form I.

The present study was prompted by the isolation of a second monoclinic polymorph of DMCA (form II) which shows distinctly different packing to the triclinic form I. The two polymorphs also show completely different photomechanical behavior. The present study addresses three issues: (i) a comparative analysis of the crystal packing of both polymorphs and the corresponding truxillic acid dimer; (ii) the relationship between the crystal packing of each form with the respective photomechanical properties as studied with nanoindentation; (iii) a comparative analysis between the rates of the reactions and the yields.

## 2. Experimental   

### 2.1. Crystallization details   

Single crystals of DMCA polymorphs were grown by slow evaporation from various solvents at room temperature. Crystals of form I in platelet morphology were obtained from a saturated MeCN or from 1:1 acetone–MeOH. The crystals are generally striated and of high mosaicity. Block-shaped crystals of form II were obtained from EtOH and MeOH. These crystals have a uniform and smooth external appearance. In some cases (1,4-dioxane, THF and EtOAc) both polymorphs were obtained concomitantly. Single crystals of the corresponding α-truxillic acid of DMCA were obtained by crystallizing the irradiated mass from MeOH.

### 2.2. Single-crystal X-ray diffraction   

Single-crystal X-ray data of form II and of the truxillic acid were collected on a Rigaku Mercury 375/M CCD (XtaLAB mini) diffractometer using graphite-monochromated Mo *K*α radiation at 150 K. The data were processed with the *CrystalClear* software (Rigaku, 2009 ▸). The structure solution was performed by direct methods, and refinements were executed using *SHELX*97 (Sheldrick, 2008 ▸) and the *WinGX* (Farrugia, 1999 ▸) suite of programs. Refinement of coordinates and anisotropic displacement parameters of non-H atoms were carried out with the full-matrix least-squares method. Face indexing of good quality single crystals of DMCA polymorphs was performed with *CrystalClear* and the major faces were assigned (see the supporting information §S1). Crystallographic CIF files (CCDC No. 1409385 and 1409386) are also available at www.ccdc.cam.ac.uk/data_request/cif.

### 2.3. Differential scanning calorimetry (DSC)   

Scans were performed on samples weighing 2 mg each in a Mettler Toledo DSC 823e instrument within the range of 25 and 300°C with a heating/cooling rate of 5°C min^−1^ in a liquid nitrogen atmosphere.

### 2.4. UV irradiation   

To reduce an effect of the crystal sizes on the reaction yields and rates, the crystalline powder materials of DMCA polymorphs were spread on the glass dish and subjected to uniform exposure of UV irradiation from a high-pressure mercury lamp with a 360 nm cutoff filter. The irradiated samples were collected after 5 and 10 h for the solution ^1^H NMR studies.

### 2.5. Solution NMR studies   

Solution-state ^1^H NMR spectra were recorded on Bruker AVANCE 400 MHz NMR spectrometers, with the samples dissolved in deuterated DMSO-d_6_ solvent. Spectra were recorded for both the polymorphs before and after the photoirradiation for specific time intervals.

### 2.6. Nanoindentation   

Large dried well shaped crystals of DMCA polymorphs were selected, after viewing them through an optical microscope supported by a rotatable polarizing stage, for the nanoindentation experiments. First, the selected crystals were firmly mounted on a stud using a thin layer of cyanoacrylate glue. Nanoindentation experiments were performed on (001) and (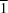
01) facets of the crystal of form I and form II, respectively (see supporting information §S1), using the Hysitron Triboindenter (Minneapolis, USA) equipped with a Berkovich tip (end radius ∼ 100 nm) at room temperature. To identify the flat and smooth regions for the experiment, the crystal surfaces were imaged prior to indentation using the same indenter tip, which also serves as an atomic force microscope (AFM). During the experiment, load (*P*) *versus* displacement (*h*) of the indenter was recorded with resolutions 1 nN and 0.2 nm, respectively. The loading and unloading rates were 0.6 mN s^−1^, and the hold time at the peak load of 6 mN was 30 s. Five to six crystals of each form were examined and a minimum of 20 indentations were performed on each crystal to ascertain the reproducibility of the data. The *P*–*h* curves obtained were analyzed using the standard Oliver–Pharr (O–P) method (Oliver & Pharr, 1992 ▸) to determine *E* and *H* of the crystals in that orientation.

### 2.7. Kinematic analysis   

The photomechanical behavior of form I crystals were investigated by selecting hand-picked good quality crystals and examining them under a microscope. The crystals were irradiated at room temperature with an ultraviolet (UV) LED torch of 375 nm with 10 degree lens and 2000 uW flashlight. The video of photomechanical response (rolling and jumping) during irradiation was recorded with a microscope equipped with a camera.

## 3. Results and discussion   

### 3.1. Crystal structure analysis   

#### 3.1.1. Form II   

**Figure 2:**
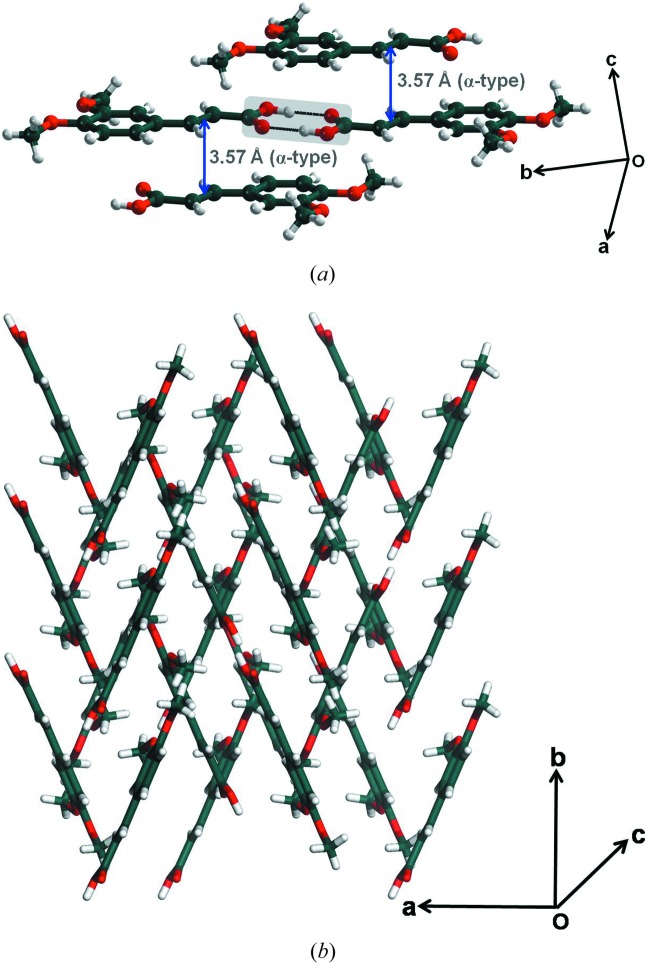
Form II of DMCA: (*a*) potentially photoreactive sites; (*b*) interlocked structure to show the absence of slip planes.

Form II of DMCA crystallizes in the space group *P*2_1_/*n* with *Z* = 4 (Table 1 ▸, Fig. 2 ▸). The primary synthon is the carboxylic acid dimer. These primary modules further interact with each other through C—H⋯O and π⋯π stacking interactions. The closest double bond to double bond mid-point distance between two molecules is 3.574 Å rendering all the DMCA molecules potentially photoreactive (Fig. 2 ▸ *a*). This is in contrast to form I wherein only half the molecules are in a potentially reactive orientation. The arrangement between two closest molecules in a stacked pair is head to tail so that the crystal is of the α-type. A closer inspection of this structure reveals that DMCA molecules are in a *syn* conformation in contrast to form I where DMCA molecules adopt an *anti*-conformation. This *syn* conformation of the DMCA molecule places a restriction on the formation of C—H⋯O interactions between carboxylic acid dimers in the same plane, as is possible in form I and many other *trans*-cinnamic acids. Because of this restriction, hydrogen-bonded carboxyl dimers in form II are connected to each other through weak out-of-plane C—H⋯O contacts leading to zigzag packing (Fig. 2 ▸ *b*). This difference in packing has major consequences on the mechanical properties of crystals of the two forms: slip planes are possible in form I, these planes in effect being constituted with planar molecules connected with in-plane O—H⋯O and C—H⋯O interactions, whereas form II lacks slip planes and packs in a more isotropic and three-dimensional manner.

DSC studies show that form I melts at 183°C while form II shows two endotherms at 164°C and 183°C, respectively, indicating an enantiotropic phase transition to form I at 164°C, and confirming that form I is the thermodynamically stable form (see supporting information §S2). This is noteworthy because it is more likely that the polymorph with the higher *Z*′ value is the more stable form (Nangia, 2008 ▸). However, the concomitant appearance of both polymorphs in certain solvents (1,4-dioxane, THF and EtOAc) hints towards a close energetic relationship between them (energy difference estimated from DSC is 1.18 ± 0.02 kJ mol^−1^).

#### 3.1.2. Truxillic acid dimer   

**Figure 3:**
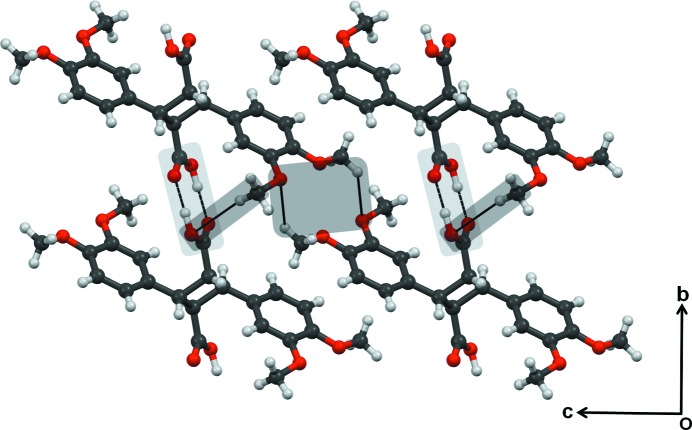
Packing diagram of truxillic acid dimer. Note the O—H⋯O hydrogen-bonded carboxylic acid dimer synthons are shaded in light gray and C—H⋯O interactions are shaded in dark gray.

The crystal structure of truxillic acid photodimer was also obtained (Table 1 ▸, Fig. 3 ▸) and it is constituted with O—H⋯O hydrogen-bonded carboxylic acid dimer synthons. This leads to the formation of one-dimensional chains which form a close-packed structure. C—H⋯O interactions also play a role in the packing, and are mainly between methoxy groups.

### 3.2. Photomechanical behavior   

#### 3.2.1. Comparison of cooperative molecular motions   

**Figure 4:**
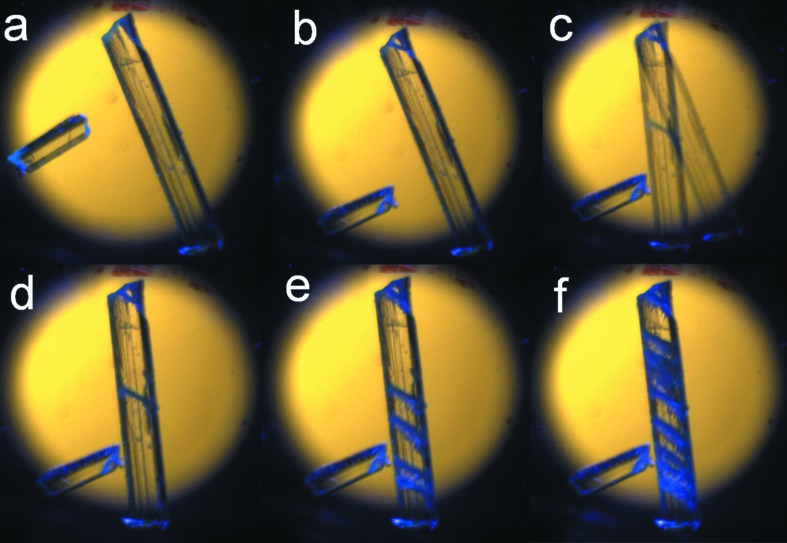
Snapshots (*a*)–(*f*) of form I crystals to a shown kinematic effect during UV irradiation. Snapshots (*d*)–(*f*) shows visible parallel cracks generated on the crystal surface.

The two crystal polymorphs show markedly different morphologies. Form I crystals are mostly plate or needle shaped, indicating anisotropy of interactions. Only block-type crystals are, however, observed for form II indicating the more isotropic packing in these crystals. This difference in crystal morphology also manifests in the photomechanical behavior of the two forms and they show strikingly different mechanical responses upon photoirradiation. While irradiated with UV light of wavelength 375 nm, cracks are quickly developed on form I crystals (see supporting information §S3) and this leads to jumping and rolling (Figs. 4 ▸ *a*–*c*) (see the video in the supporting information).

**Figure 5:**
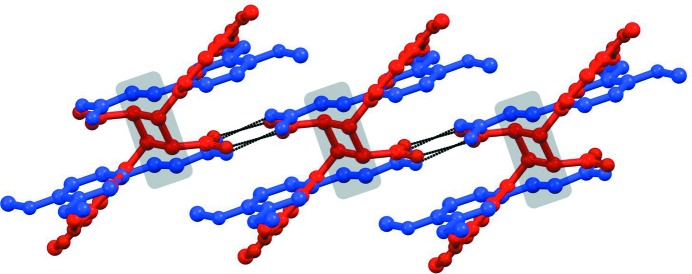
An overlap diagram of form II (blue color) and truxillic acid dimer (red color).

A comparative analysis of the crystal structures of both forms I and II with that of the truxillic acid dimer sheds further insights into the mechanism of this photo-transformation. It is observed that the mutual disposition of molecules in the photoproduct is closer to the crystal packing of form II when compared with that in form I. Fig. 5 ▸ is an overlap diagram of form II and the dimer crystal structures and illustrates this idea.

**Figure 6:**
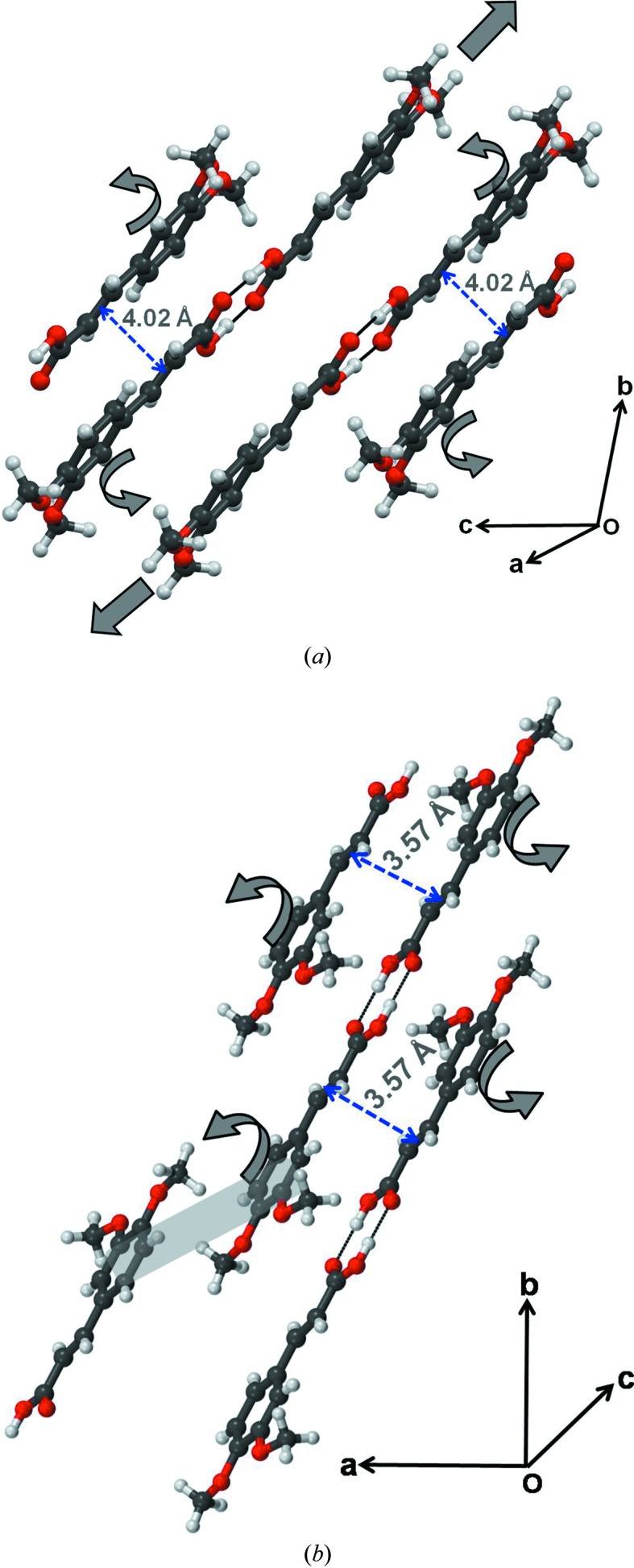
A pictorial depiction of the probable mechanism of photoreaction in (*a*) form I and (*b*) form II. Arrows represent the directions of movement of molecules during photochemical reaction.

If this crystal structure of the (recrystallized) photoproduct bears any relationship to that of the truxillic acid as it is formed *in situ* in the photoreaction, it would suggest that photodimerization of form II crystals takes place with a minimum of molecular movement and that conversely, photodimerization in form I requires more molecular movement. Under such an assumption, it can also be concluded from Fig. 5 ▸ that there is hardly any movement of the O—H⋯O hydrogen-bonded synthons during the photo-transformation. Of course, half the molecules in the form I crystal are not even organized for a solid-state photoreaction so that it may well be concluded that rather major molecular movements are involved in its photoreaction considering the yield is even as high as 40% (see §3.3). The observation that cracks appear in a perpendicular direction to the main elongation axis of form I may thus be rationalized on the basis of molecular movements as shown in Fig. 6 ▸(*a*). In contrast, photoreactivity in form II needs only minor adjustments of the structural features (Fig. 6 ▸ *b*).

**Figure 7:**
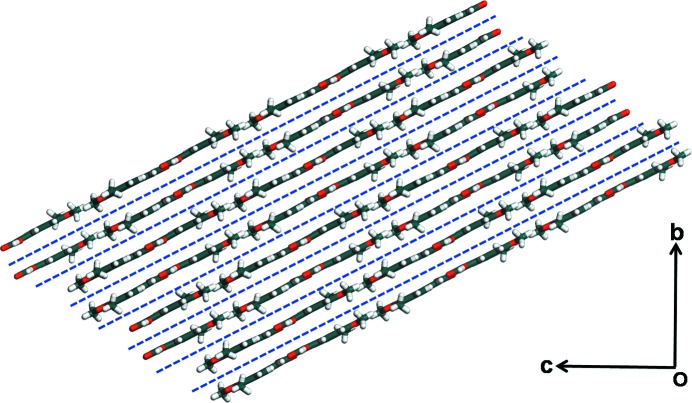
Molecular packing of DMCA form I. Blue dotted lines in form I represent slip planes.

The appearance of parallel cracks in the direction [011] towards the main elongation axis (*b* axis) of the crystal is also indicative of significant molecular movements within form I crystals upon photoirradiation (Figs. 4 ▸ *a*–*f*). The build-up of strain due to such molecular movement is released through cleavage of molecular planes; this is in itself facilitated by the presence of slip planes in form I across which there is considerable space for molecular movement (Fig. 7 ▸). Cleavage leads to a sudden release of accumulated strain in the form of kinetic energy. All this results in the observed photosalient behavior (Medishetty *et al.*, 2014 ▸, 2015 ▸; Nath *et al.*, 2014 ▸; Kim *et al.*, 2014 ▸). In contrast, the crystal structure of form II is interlocked and this restricts such movement of molecules (Fig. 2 ▸ *b*). The observed jumping behavior in form I and absence of it in form II is indicative of faster reaction in form I during the initial stages when the integrity of the slip planes is maintained in the pristine reactant crystal.

#### 3.2.2. Nanoindentation   

In order to explain the physical origin of the photosalient effect, the mechanical properties of the major face of both the crystal forms were evaluated with the nanoindentation technique.

**Figure 8:**
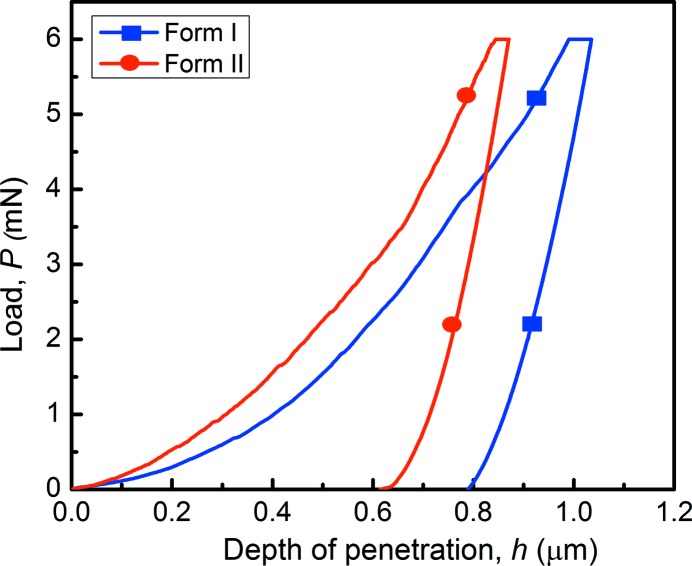
Representative *P*–*h* curves of the DMCA forms.

Representative load (*P*) *versus* depth of penetration (*h*) responses for both the forms are shown in Fig. 8 ▸. From these, *E* and *H* values are extracted, which were 8.42 ± 0.28 GPa and 270.0 ± 5.9 MPa, respectively, for form I, and 9.57 ± 0.13 GPa and 390.7 ± 1.5 MPa, respectively, for form II. These results indicate that form II is more stiffer and harder than form I. The difference in *H* (∼31%) is more than *E* (∼12%). Here it is instructive to briefly mention as to what these properties reflect. The resistance offered by a material to elastic deformation can be measured through *E*. In molecular crystals, it depends on crystal packing, the strength of intermolecular interactions and their orientation with respect to the indentation direction (Varughese *et al.*, 2013 ▸). *H*, on the other hand, is a measure of the indented material’s resistance to plastic (or permanent) deformation, and depends on the relative ease with which molecular layers can slide irrecoverably past each other upon the application of stress. Thus, the observation of only a marginal difference in *E* in these two forms is due to the presence of similar interactions in both forms. However, the interlocked structure and interactions in all three directions of form II makes it considerably harder (as there are no slip planes that would allow for gliding of molecular planes past each other under the application of stress). This lack of plastic deformability in form II makes it brittle compared with form I. In summary, the availability of the slip plane in form I (blue dotted in Fig. 7 ▸) makes it softer than form II that allows greater molecular movement upon irradiation. Taking into account that form I reacts faster than form II during the initial stages of the photochemical reaction and correlating it with the nanoindentation results, it can be said that plasticity favors maximum movements within crystals.

### 3.3. Maximum yields and reaction rates   

Two factors are important in any consideration of the mechanism of these solid-state reactions: the initial and final rates of reactions, and the maximum yields. Analysis of the crystal packing of both the DMCA forms and the corresponding truxillic acid dimer, their photomechanical behavior and nanoindentation results indicate faster initial reaction in form I but with the maximum yield being restricted to around 40% as molecules ‘go beyond’ the topochemical limit in the later stages; notably, the maximum yield does not touch the ‘ideal’ 50%.^1^ On the other hand, form II is brittle and the packing is dominated by isotropic interactions. The reaction is slower initially but the reactive monomer molecules are always within the topochemical limit, and so the maximum yield is relatively high. This is reflected in a 31% yield after 5 h of irradiation that ultimately leads to 85% yield after 10 h.^2^ The rate of reaction is higher for form I after 5 h (34%), but the final yield after 10 h is lower (78%) (see supporting information §§S4 and S5). This observation suggests that photoreaction in form I is dictated by initial surface reaction which is fast but there is a decay in the rate in the later stages because of a breakdown in the original (topochemically permissible) crystal packing. On the other hand, in form II photoreaction is more faithfully governed by topochemical rules because gross molecular movement is more difficult. The initial reaction rate is slow because the structure is interlocked but proceeds to high conversions, again because the structure is interlocked and the dimer structure is topologically similar to that of the reactant monomer. Our results provide a good indication that both Kaupp and Schmidt type mechanisms operate in these solid-state 2 + 2 photodimerization reactions of alkenes. Where molecular movement is facile, initial topochemically allowed photoreaction results in gross changes in mutual dispositions leading to slower reaction and lower final yields. This is the case in the triclinic form I of DMCA. On the other hand, the photoreaction in the monoclinic form II is governed more by Schmidt-type topochemistry. The reaction may be slower throughout but final yields are higher.

## 4. Conclusions   

The above example provides us with a unique opportunity to compare the two viewpoints related to the photochemistry of the molecules in the solid state. Molecular crystals can be plastic, elastic or brittle. Taking all the observations into account, it can be said that either or both Schmidt-type or Kaupp-type mechanisms are valid. As Schmidt’s hypothesis of minimum molecular movement in the solid-state topochemical reactions restricts the molecules in their original positions, brittle and elastic solids may be the best for this type of topochemistry. The crystals with such mechanical properties show interaction isotropy within crystals that restrict molecular movements (as observed in form II). On the other hand, plastic solids may be best for the Kaupp-type photoreactions as they have the required anisotropy for the molecular movements. It is only possible now, with the aid of the nanoindentation technique, to correlate these photochemical mechanisms with the mechanical behavior of the crystal structures themselves. It is expected that this technique may open up new avenues for the design of functional photoactive systems with desired mechanical properties in the near future. This system also opens up new vistas in photocrystallography.

## Supplementary Material

NMR spectra of compounds. DSC of polymorphs. SEM images. Face Indexing details.. DOI: 10.1107/S2052252515017297/lc5066sup6.pdf

Video of photomechanical behavior of form I. DOI: 10.1107/S2052252515017297/lc5066sup5.avi

Crystal structure: contains datablock(s) dmcaformII, DMCAtruxillicacid. DOI: 10.1107/S2052252515017297/lc5066sup1.cif

Structure factors: contains datablock(s) dmcaformII. DOI: 10.1107/S2052252515017297/lc5066dmcaformIIsup2.hkl

Structure factors: contains datablock(s) DMCAtruxillicacid. DOI: 10.1107/S2052252515017297/lc5066DMCAtruxillicacidsup3.fcf

Supporting information file. DOI: 10.1107/S2052252515017297/lc5066sup4.mp4

CCDC references: 1409385, 1409386

## Figures and Tables

**Table 1:** Crystallographic details of form II and its truxillic acid dimer

	Form II	Truxillic acid
Crystal data		
Chemical formula	C_11_H_12_O_4_	C_22_H_24_O_8_
*M*_r_	208.21	416.41
Crystal system, space group	Monoclinic, *P*2_1_/*c*	Triclinic, 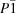
Temperature (K)	150	150
*a*, *b*, *c* ()	11.216(6), 8.214(6), 14.073(12)	5.540(4), 8.259(5), 11.281(9)
, , ()	90, 128.86(3), 90	83.61(2), 83.275(17), 74.48(2)
*V* (^3^)	1009.6(13)	492.2(6)
*Z*, *Z*	4, 1	1, 0.5
Radiation type	Mo *K*	Mo *K*
_calc_ (gcm^3^)	1.370	1.405
*F*(000)	440.0	220.0
(mm^1^)	0.11	0.11
range for data collection ()	2.927.5	1.827.5
Crystal size (mm)	0.25 0.15 0.10	0.25 0.20 0.10

Data collection		
Diffractometer	Rigaku Mercury375R (22 bin mode)	Rigaku Mercury375R (22 bin mode)
Absorption correction		
No. of measured, independent and observed [*I* > 2(*I*)] reflections	10242, 2304, 2083	5242, 2251, 2019
*R*_int_	0.063	0.040
(sin /)_max_ (^1^)	0.650	0.651

Refinement		
*R*[*F* ^2^ > 2(*F* ^2^)], *wR*(*F* ^2^), *S*	0.043, 0.127, 1.08	0.038, 0.123, 1.12
No. of reflections	2304	2251
No. of parameters	138	172
H-atom treatment	H atoms treated by a mixture of independent and constrained refinement	H atoms treated by a mixture of independent and constrained refinement
_max_, _min_ (e ^3^)	0.24, 0.32	0.30, 0.23
CCDC No.	1409385	1409386

## Footnotes

1 Yields of the reaction were estimated by taking solution NMR spectra after 5 and 10 h of irradiation.

2 It must be noted here that as only 50% of molecules are photoreactive in form I, the % yield (and the respective rate) is doubled for form I in order to carry out a comparative analysis with form II.
