# Supplementary material for: Crystal chemistry and photomechanical behavior of 3,4-dimethoxycinnamic acid: correlation between maximum yield in the solid-state topochemical reaction and cooperative molecular motion
Source: IUCrJ. 2015 Oct 16;2(Pt 6):653–60. doi: 10.1107/S2052252515017297 (PMC4645110; doi:10.1107/S2052252515017297)
Supplement: Supplementary file 1 [file m-02-00653-sup6.pdf]

IUCrJ

Volume 2 (2015)

Supporting information for article:

Crystal chemistry and photomechanical behavior of 3,4-dimethoxycinnamic acid: correlation between maximum yield in the solid-state topochemical reaction and cooperative molecular motion

Manish Kumar Mishra, Arijit Mukherjee, Upadrasta Ramamurty and Gautam R. Desiraju

Figure S1 Face index images of DMCA polymorphs

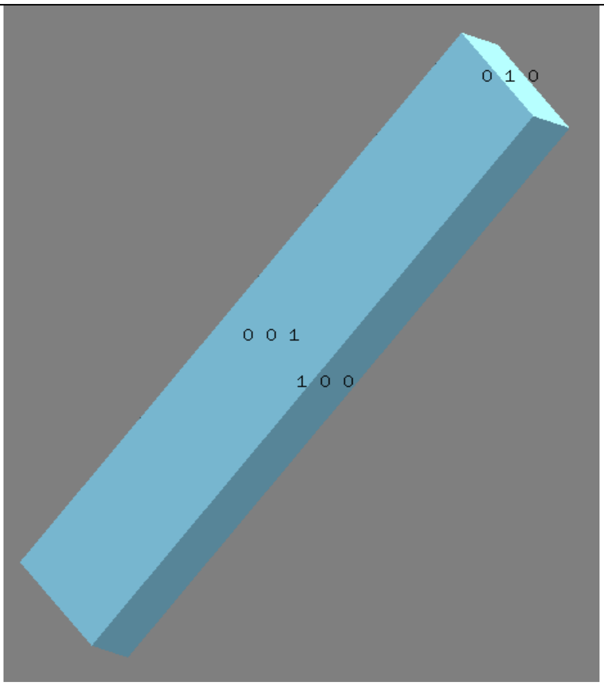	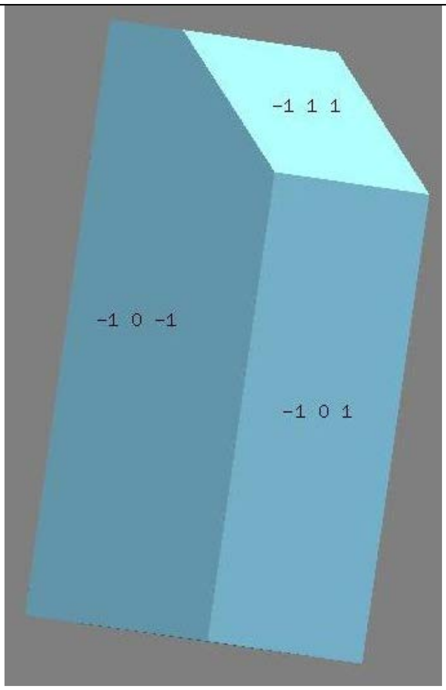
**Form I**	**Form II**

Figure S2 DSC and melting point (m.p.) of DMCA polymorphs and truxillic acid dimer

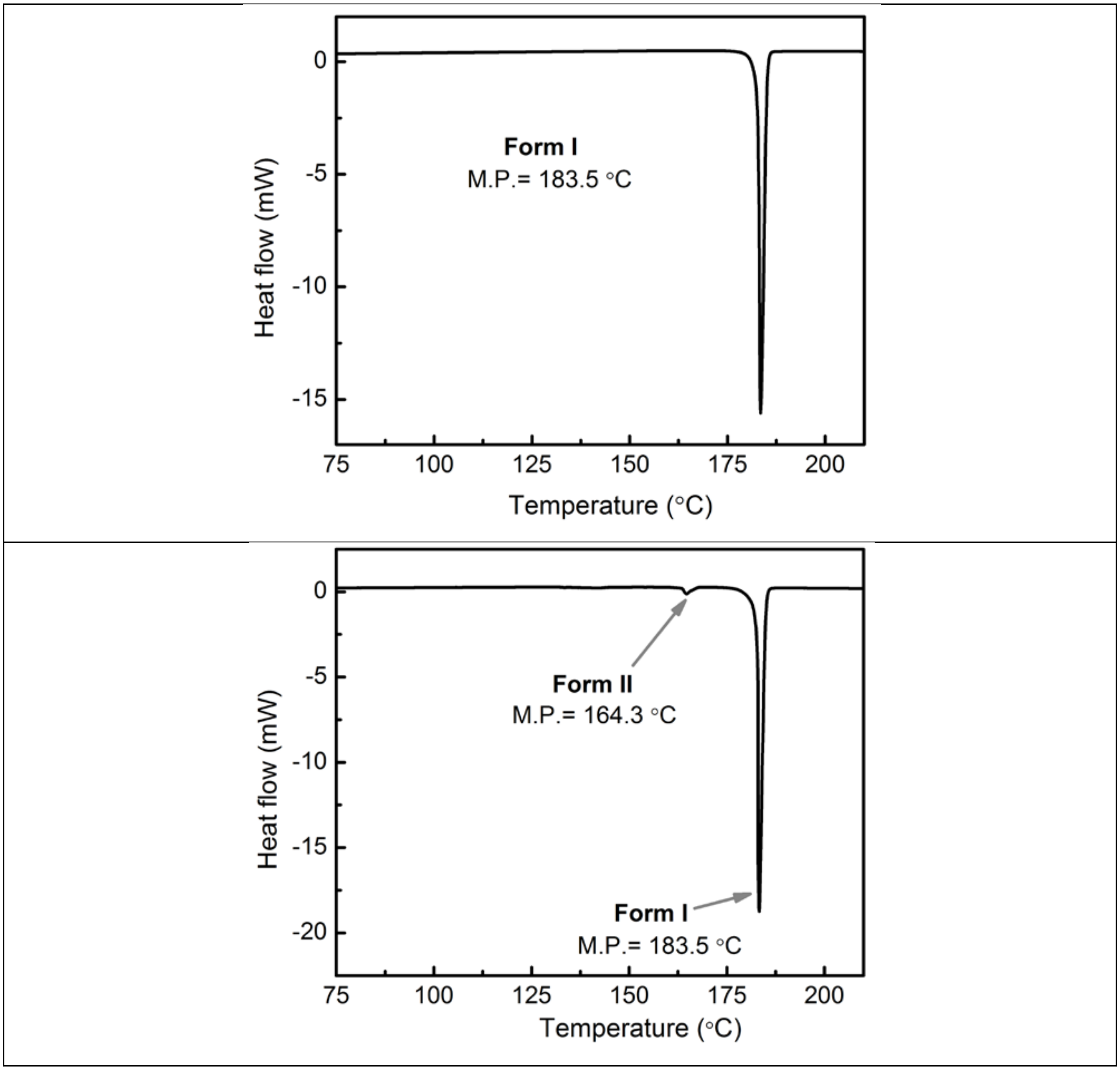

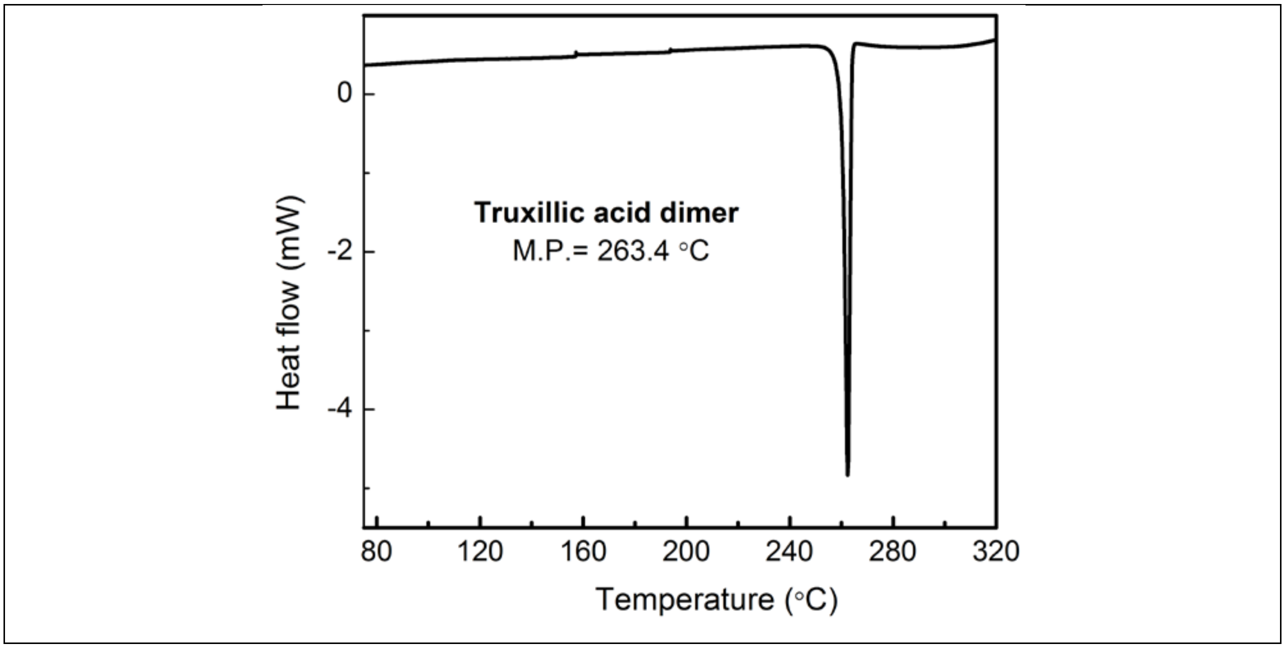

Figure S3 SEM images of the cracks on the crystal surface (001) face of form I

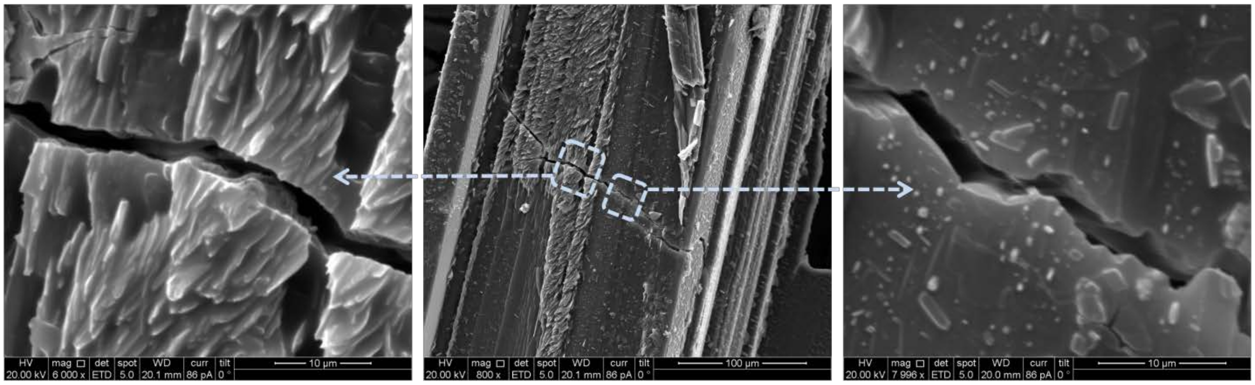

Figure S4 Solution state ^{1}H NMR spectra recorded for form I (a) and form II (b) of DMCA polymorphs after UV irradiation for 0, 5 and 10 hr.

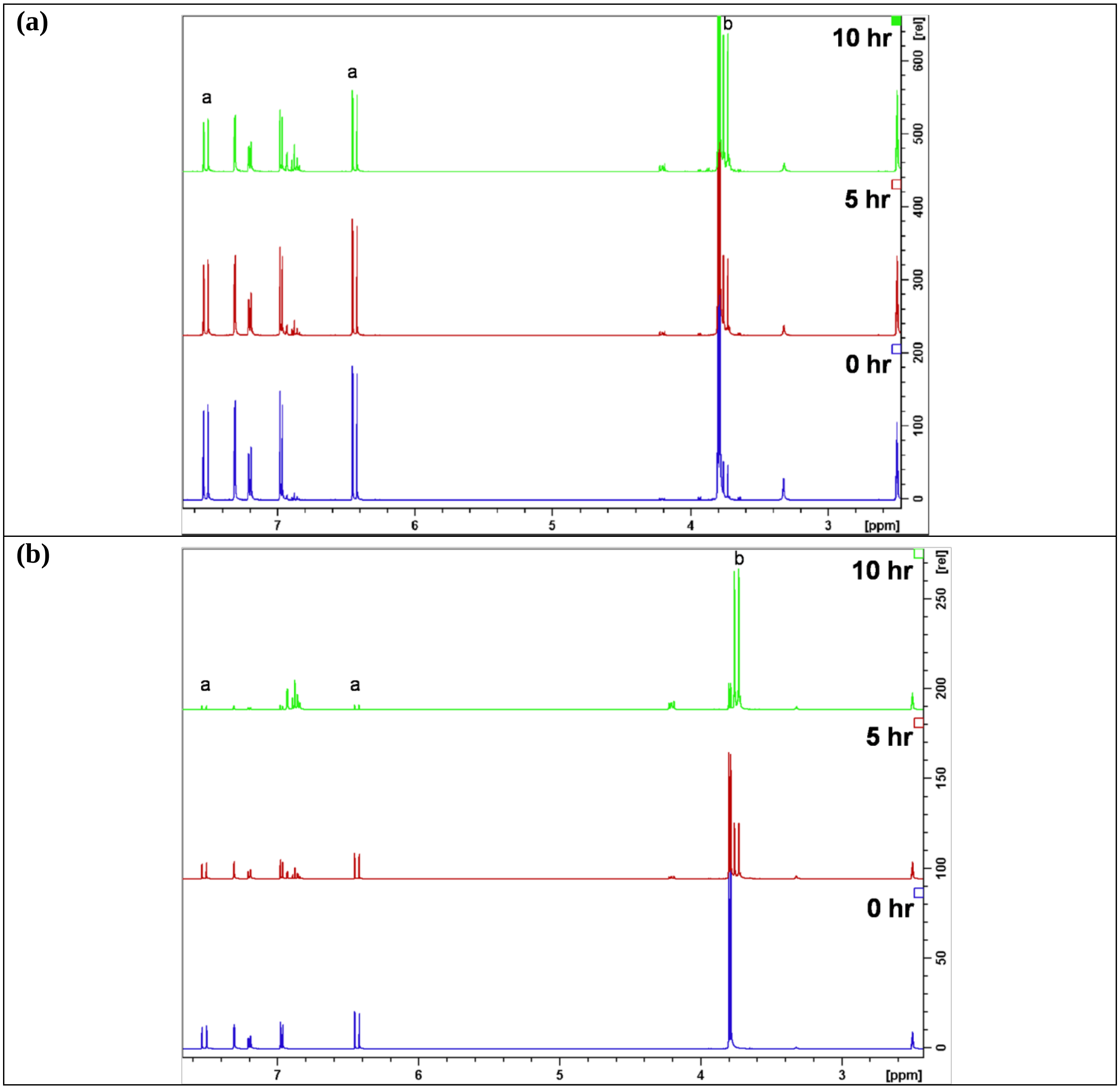

Figure S5 NMR spectra of DMCA polymorphs

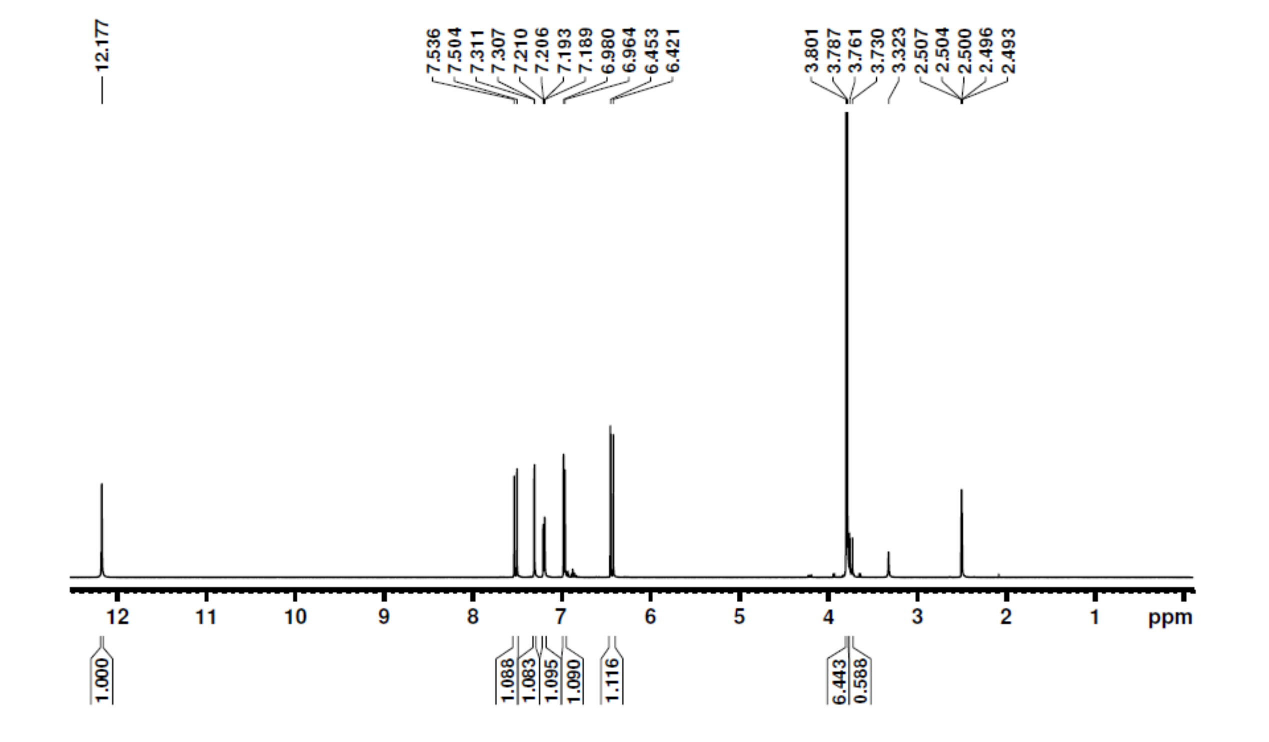

Form I (0 hr)

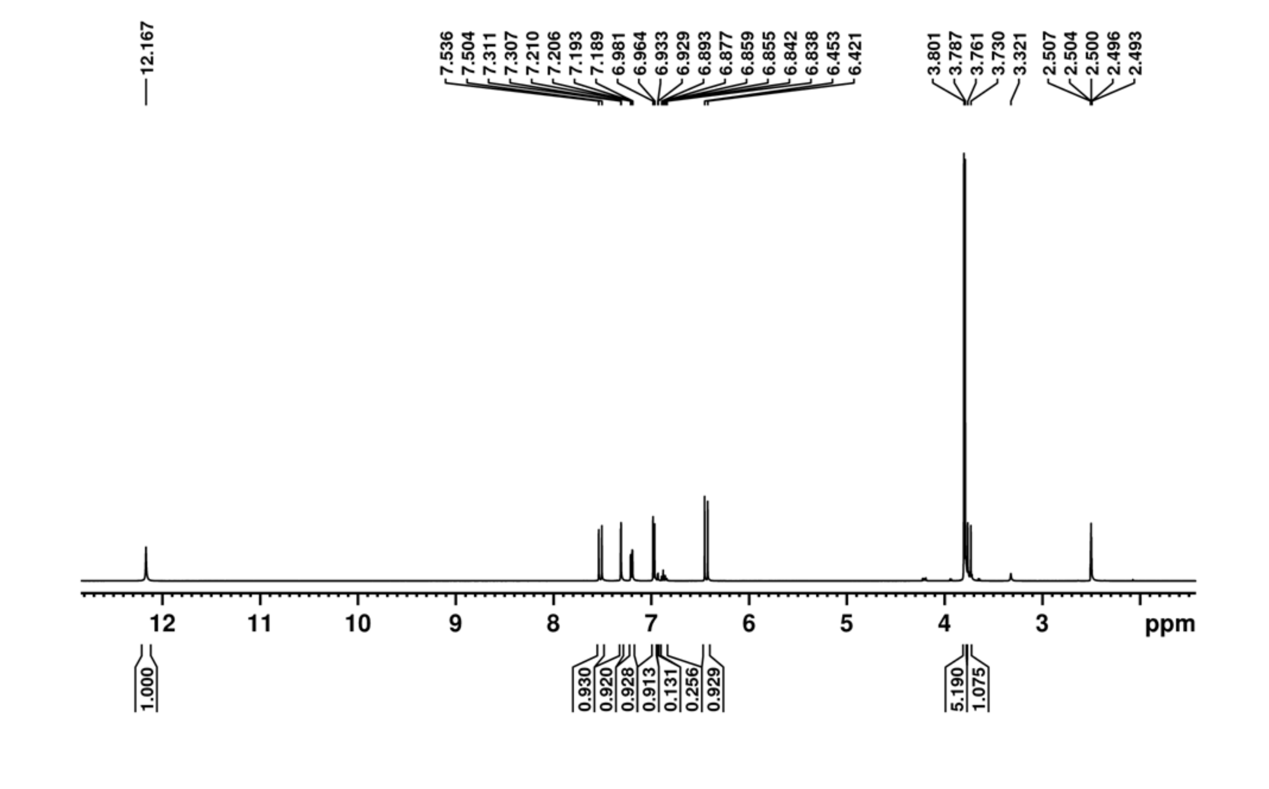

Form I (5 hr)

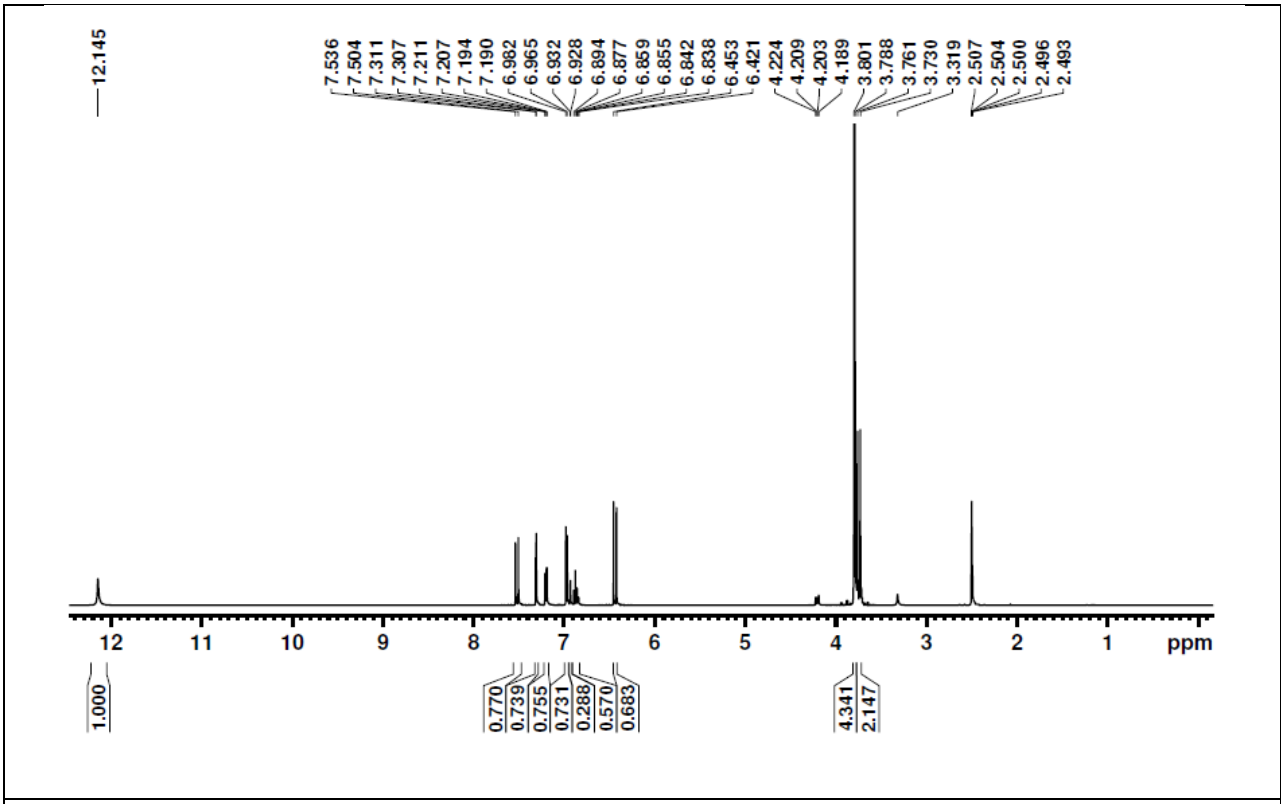

Form I (10 hr)

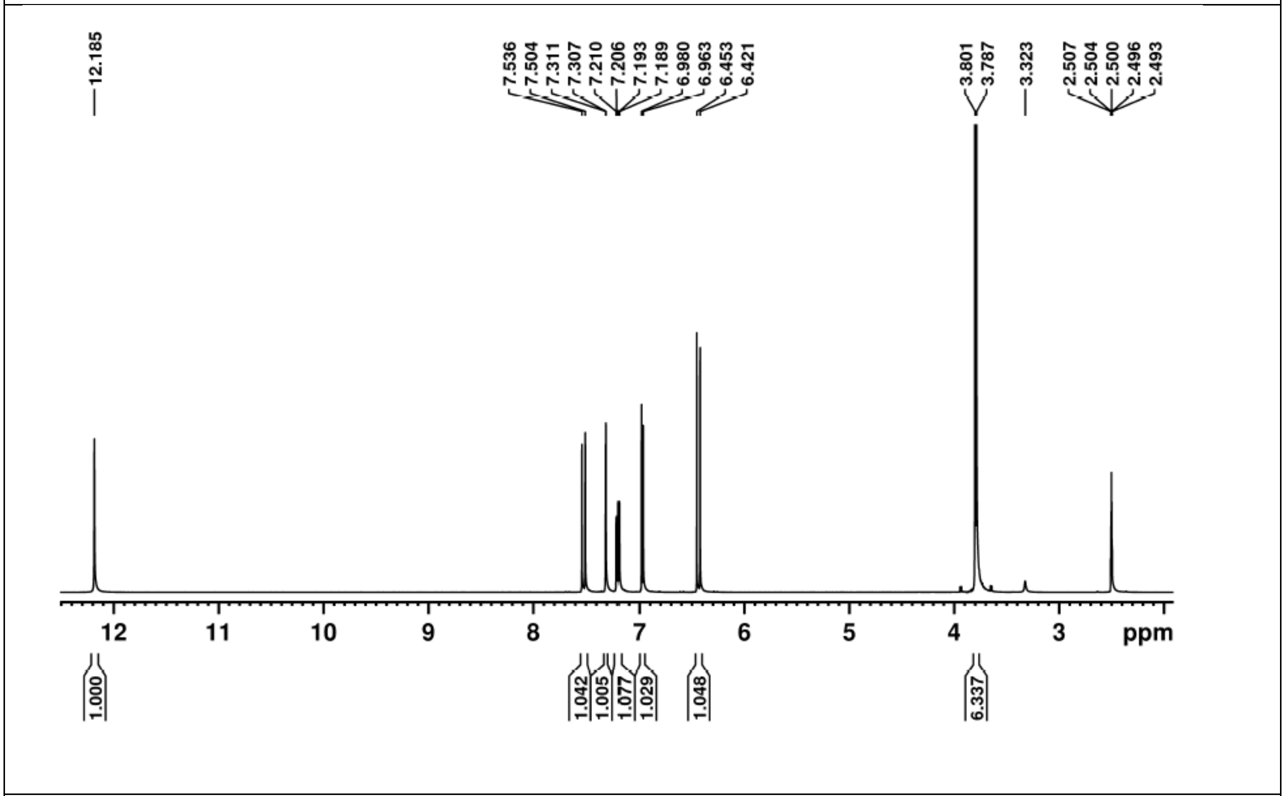

Form II (0 hr)

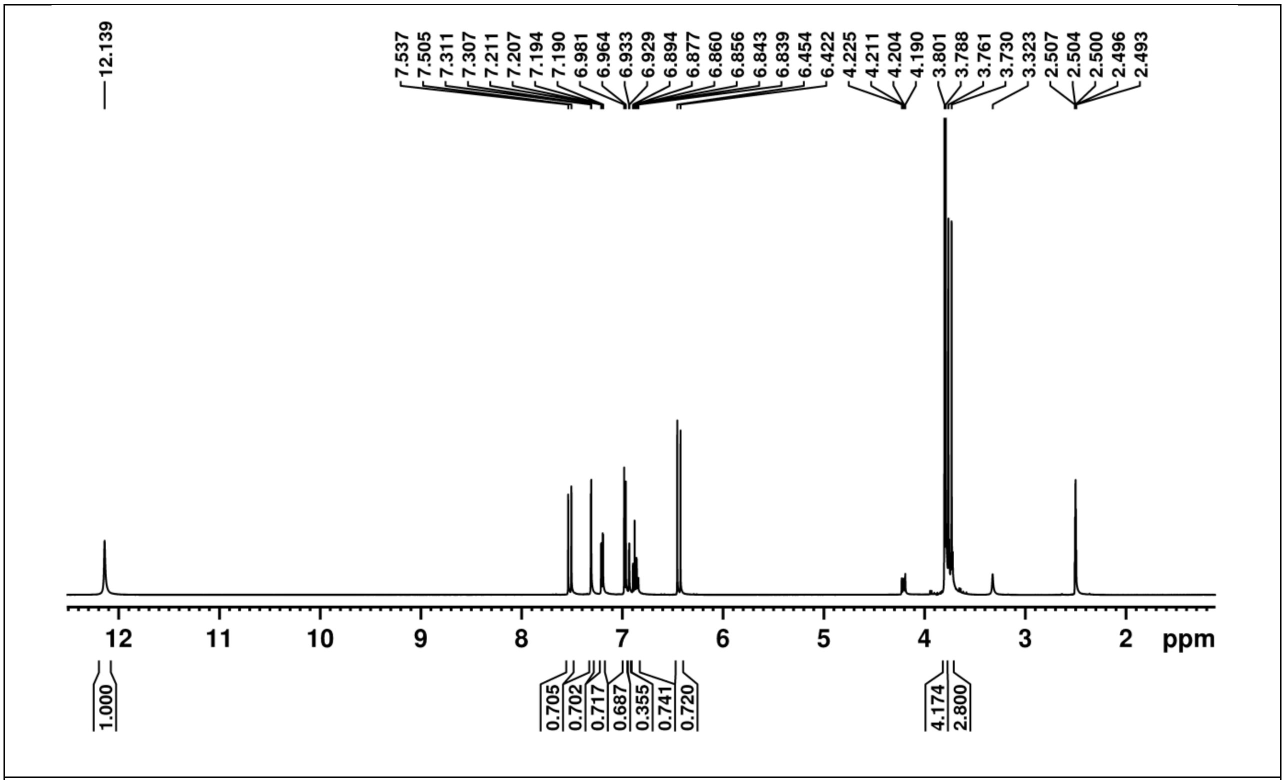

Form II (5 hr)

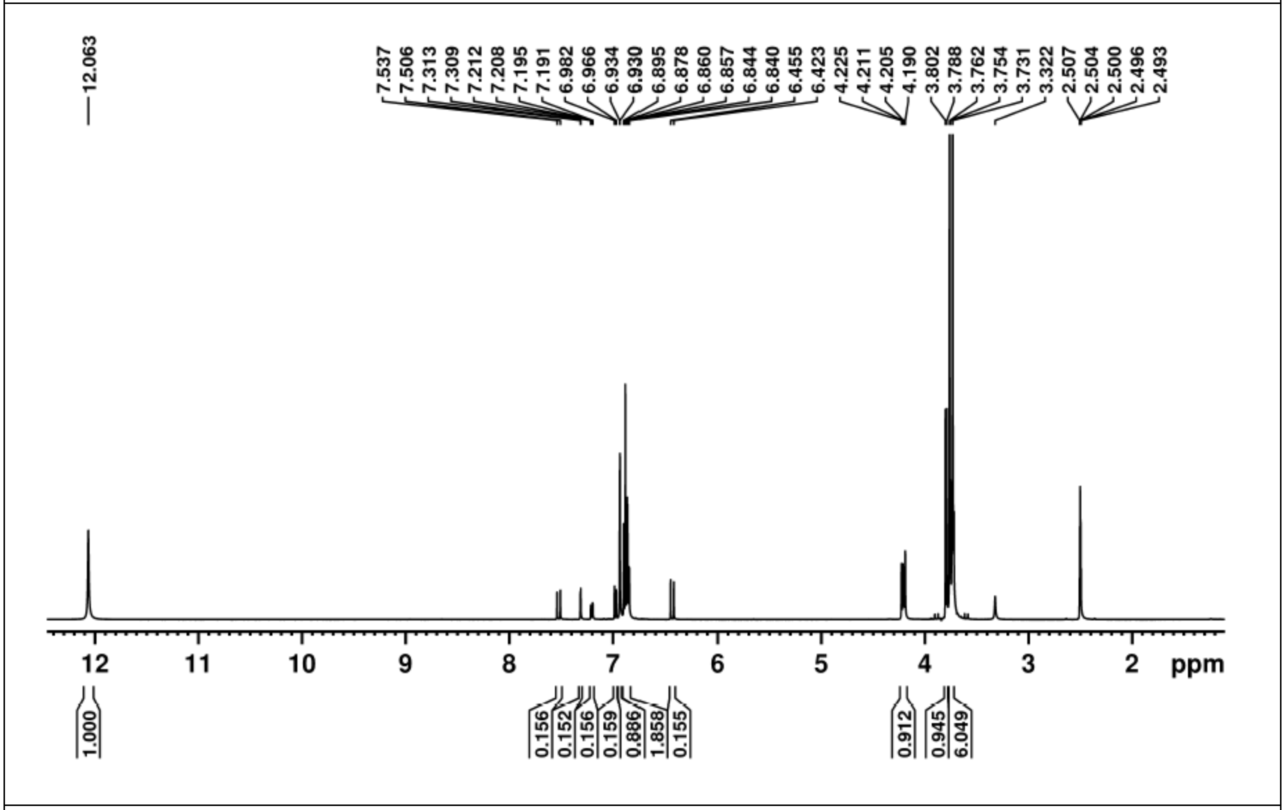

Form II (10 hr)
